# Uncovering the gut - skin axis: the role of specific traditional Chinese medicine interventions in regulating gut microbiota for diabetic foot ulcers and the analysis of research status

**DOI:** 10.3389/fphar.2025.1641036

**Published:** 2025-09-25

**Authors:** Nian Zhou, Liuju Shi, Xiangke Yuan, Jingyu Lang, Jixue Wang, Jianpeng Li, Yibo Wang, Yanan Li

**Affiliations:** ^1^ Henan Provincial Hospital of Traditional Chinese Medicine (The Second Affiliated Hospital of Henan University of Traditional Chinese Medicine), Zhengzhou, Henan, China; ^2^ Henan University of Traditional Chinese Medicine, Zhengzhou, Henan, China; ^3^ Zhengzhou Seventh People’s Hospital, Zhengzhou, China; ^4^ Heilongjiang University of Chinese Medicine, Harbin, China; ^5^ The First Affiliated Hospital of Henan University of Traditional Chinese Medicine, Zhengzhou, Henan, China

**Keywords:** traditional Chinese medicine, gut microbiota, diabetic foot ulcers, acupuncture, SCFAs, botanical drugs

## Abstract

Diabetic foot ulcers (DFU), a severe complication of diabetes, are closely linked to gut-skin axis dysregulation, including gut microbiota imbalance, systemic inflammation, and impaired skin barrier function. This review highlights the potential of specific TCM interventions, including special traditional Chinese medicine preparations and acupuncture, in modulating this axis to treat DFU. The botanical drugs (e.g., *Astragalus membranaceus* Bunge (Milkvetch root, Fabaceae; official drug name: Huangqi), *Paeonia lactiflora* Pall. (Peony root, Paeoniaceae; official drug name: Baishao) and botanical drugs formulas (e.g., Jinhuang Powder, Simiao Yong’an Decoction) regulate gut microbiota to increase short-chain fatty acids (SCFAs), reduce pro-inflammatory cytokines (IL-1β, TNF-α), and enhance intestinal barrier integrity via tight junction proteins (ZO-1, claudin-1). Acupuncture, through techniques like encircling needling and moxibustion, improves microcirculation in lower limbs, activates the vagus nerve-anti-inflammatory pathway, and promotes SCFA production to alleviate inflammation and accelerate wound healing. Mechanisms involve multi-target regulation of Wnt/β-catenin, PI3K/AKT, and Nrf2 signaling pathways to enhance angiogenesis, collagen synthesis, and epidermal stem cell proliferation. The ability of special traditional Chinese medicine preparations and acupuncture to solve intestinal microbiota imbalance and skin repair provides a novel comprehensive strategy for DFU management, which is worth conducting large-scale clinical trials to verify its efficacy and safety. This review also evaluates the current evidence gaps, including small sample sizes in clinical trials and inconsistent preparation standards, which need to be addressed in future research.

## 1 Introduction

Diabetic foot ulcers (DFU) are one of the common and severe complications in diabetic patients, characterized by high incidence and morbidity rates. DFU not only severely reduces patients’ quality of life but also increases the medical economic burden. Statistical data show that approximately 15%–25% of diabetic patients worldwide will experience foot ulcer problems, and these patients have a significantly increased risk of amputation (Jifar et al., 2021). The gut-skin axis refers to the bidirectional communication network linking intestinal microbiota, immune responses, and skin homeostasis. Dysregulation of this axis—characterized by gut microbial imbalance, increased intestinal permeability, systemic inflammation, and impaired skin barrier function—contributes to DFU pathogenesis by exacerbating tissue damage and delaying wound healing (Mahmud et al., 2022). Recent studies have identified the gut-skin axis as a critical factor in the occurrence and progression of DFU. Dysregulation of the gut microbiota, enhanced inflammatory responses, and diminished skin barrier function are all closely associated with the progression of foot ulcers (Ye et al., 2022; Liu et al., 2022). Special traditional Chinese medicine preparations (STCMP) and acupuncture, as conventional therapeutic approaches, have demonstrated potential in modulating gut microbiota, alleviating inflammation, and promoting skin healing, thereby providing new insights and strategies for DFU intervention (Dai et al., 2022; Liang et al., 2024). In this article, special traditional Chinese medicine preparations include single botanical drugs and their extracts, metabolites, and formulas composed of multiple botanical drugs. Specific dosage forms include decoctions, pills, powders, and ointments.

In the treatment of DFU, the application of TCM has gained increasing attention. Studies have demonstrated that botanical drugs such as *Astragalus membranaceus* Bunge (Milkvetch root, Fabaceae; official drug name: Huangqi) and *Rehmannia glutinosa* (Gaertn.) Libosch. ex Fisch. et Mey. (Chinese foxglove root, Orobanchaceae; official drug name: Dihuang) can promote blood circulation, improve microcirculation, and enhance immune function, effectively alleviating clinical symptoms in DFU patients (Fan S. et al., 2021; Zhang Z. et al., 2019). In the treatment of DFU, traditional Chinese medicine (TCM) has built a bridge between traditional pharmacology and modern pharmacology. In a multicenter, randomized, positive controlled clinical trial, Sun et al. found that Shengji Ointment combined with bromelain could significantly promote the formation of tendon granulation in diabetes foot ulcers. The treatment group not only had a higher coverage of granulation tissue than the control group, but also significantly better wound healing rate, granulation formation time, Maryland foot function score, necrotic tendon tissue debridement time, and granulation tissue score than the control group (Sun X. et al., 2024). For neuropathy, acupuncture can improve local blood flow and nerve function by stimulating specific points, thus improving the neuropathic pain of diabetes patients, which also shows that acupuncture is effective as an auxiliary treatment for diabetes feet (Heidari et al., 2023; Lee et al., 2020). On the other hand, acupuncture may also influence the pathological process of DFU by modulating the composition of gut microbiota and improving metabolic status. Existing research has shown that acupuncture significantly improves microcirculation in diabetic patients, providing a theoretical basis for its application in DFU treatment (Valentini et al., 2024).

This review aims to investigate the mechanisms of action of STCMP and acupuncture in modulating the gut-skin axis and its application strategies in the intervention of diabetic foot ulcers. By analyzing relevant literature, we hope to provide new perspectives for the comprehensive treatment of DFU, integrating the advantages of modern medicine and TCM to promote patient recovery and quality of life improvement (Bragg et al., 2024; Huang et al., 2025).

## 2 Methods

### 2.1 Search strategy

To identify published studies, we conducted a comprehensive search of PubMed and Embase databases, covering records from January 2010 to January 2025. Our search approach comprises the following sets of keywords: [“Traditional Chinese Medicine” or “TCM” or “botanical drug” or “herb” or “herbal extracts” or “Chinese herbal formulas”], [“Diabetic foot ulcers” or “DFU” or “Diabetic wounds”], [“immune regulation”], [“Gut microbiota” or “Gut microflora” or “Gut microbiota metabolites”]. We limited our search to English publications, and the initial screening was carried out using the search engines integrated into each database.

### 2.2 Data extraction and synthesis

Before reviewing the complete content of any paper, we manually select references related to the topic using Excel. Finally, all included materials are peer reviewed articles related to the topic. When drafting the paper, one author is responsible for extracting data. Afterwards, other authors cross validated the extracted data to ensure its integrity and reliability.

## 3 Pathological mechanism of diabetes foot ulcers

**FIGURE 1:**
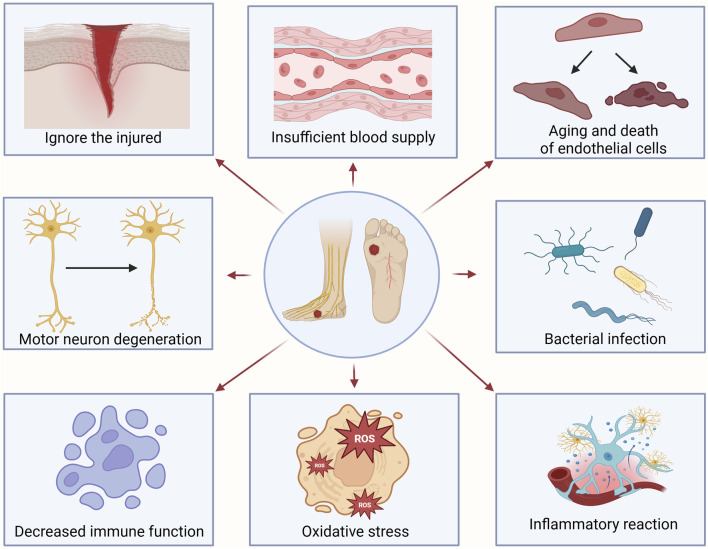
The main mechanism of diabetic foot ulcers occurrence. Ignoring the injured leads to the inability to treat them in a timely manner; Vascular disease leads to insufficient blood supply to the feet, obstruction of nutrient delivery, and weakened tissue repair ability; Degeneration of motor nerves leads to decreased skin sensitivity, uneven foot pressure load, and ultimately skin rupture and ulcers in patients; Bacterial infection triggers inflammation and exacerbates tissue damage; The aging and death of endothelial cells lead to dry and cracked foot skin; Immune system decreased leads to macrophages being unable to effectively clear necrotic tissue from wounds; Oxidative stress exacerbates inflammatory response and delays tissue repair; Inflammatory response exacerbates wound lesions and affects wound healing.

DFU are caused by various risk factors, such as peripheral neuropathy, peripheral vascular disease, foot deformities, arterial insufficiency, metabolic disorders, dysbiosis, inflammation, trauma, and reduced anti-infective capacity (Lao et al., 2019). Following DFU onset, wounds often exhibit prolonged non-healing, and the underlying pathogenesis is illustrated in Figure 1. Existing studies have shown that the development of DFU is closely associated with metabolic disorders, particularly the hyperglycemic state caused by poor glycemic control (Wang KX. et al., 2024). Hyperglycemia induces multiple metabolic abnormalities, including insulin resistance, lipid metabolism disorders, and protein synthesis dysfunction, which can all impair the normal function of microcirculation (Wang KX. et al., 2024; Raja et al., 2023). Research indicates that microcirculatory dysfunction in diabetic patients is a critical factor contributing to DFU. Damage to microvessels leads to ischemia and hypoxia in foot tissues, thereby compromising skin healing capacity and triggering ulcer formation (Liang et al., 2024). Additionally, microcirculatory dysfunction exacerbates inflammatory responses, further worsening tissue damage (Ye et al., 2022). Therefore, improving microcirculatory function and restoring blood supply to the feet represent one of the key strategies in DFU treatment.

Changes in the gut microbiota are also closely associated with the immune function of diabetic patients, as intestinal microbes can influence diabetic complications by regulating immune responses (Yuan et al., 2022). The immune system of diabetic patients is often suppressed, a change that renders them more susceptible to infections and impairs healing after infection (Fan S. et al., 2021). Studies have found that leukocyte function is weakened in diabetic patients, particularly the functions of macrophages and T cells, leading to reduced resistance to infection (Sun S. et al., 2024). Additionally, diabetes induces a state of chronic low-grade inflammation, which further suppresses immune responses and affects the wound healing process (Fu et al., 2022). Therefore, enhancing the immune function of diabetic patients and improving their resistance to infection represent critical strategies in the prevention and treatment of DFU.

Gut microbiota dysregulation can lead to increased metabolic endotoxins, thereby triggering systemic inflammatory responses, which are recognized as one of the key pathological mechanisms of DFU (Zhang K. et al., 2024). Diabetic patients often experience chronic low-grade inflammation, a state that delays wound healing and increases the risk of complications (Huang et al., 2025). Studies indicate that the inflammatory response in DFU is primarily driven by abnormal activation of macrophages and other immune cells, leading to excessive release of inflammatory mediators that impair wound healing (Lao et al., 2019). Additionally, inflammation may induce cell apoptosis and tissue damage, further exacerbating the condition. Therefore, controlling inflammatory responses is regarded as a critical strategy for improving DFU healing, and related clinical studies are continuously exploring effective anti-inflammatory treatment strategies (Wang KX. et al., 2024).

## 4 The relationship between intestinal microbiota and DFU

The gut microbiota is composed of diverse microorganisms, including bacteria, fungi, and viruses. Bacteria are the most dominant component of the gut microbiota, with common phyla including Firmicutes, Bacteroidetes, and Actinobacteria. Different microbial species perform various metabolic functions in the intestine, such as fermenting undigested food residues, synthesizing short-chain fatty acids (SCFAs), and producing vitamins (Luo et al., 2024). SCFAs like acetic acid, propionic acid, and butyric acid play critical roles in anti-inflammation, immune regulation, and maintenance of intestinal barrier function (Patel et al., 2022). Additionally, gut microbiota is involved in drug metabolism, influencing the efficacy and toxicity of medications (Wang J. et al., 2024). Therefore, the composition and metabolic functions of the gut microbiota are vital for host health.

The gut microbiota exhibits dynamic characteristics, with its composition and function influenced by multiple factors such as diet, environment, age, and disease state. Studies have shown that the gut microbiota undergoes significant changes throughout an individual’s lifecycle, particularly during infancy and the stage of introducing solid foods, where microbial diversity and composition shift remarkably (Du et al., 2023). Additionally, the stability of the gut microbiota is a critical research area. A healthy gut microbiota typically demonstrates high stability, capable of resisting external disturbances such as antibiotic use or dietary changes. However, dysregulation of the gut microbiota may lead to the development of various diseases, including diabetes, obesity, and intestinal inflammation (Zhang K. et al., 2024; Du et al., 2023; Cai et al., 2024). Therefore, a deep understanding of the dynamic changes and stability of the gut microbiota is of great significance for developing new intervention strategies and therapeutic approaches.

The microbial profiles of DFU patients differ significantly from those of healthy individuals. Studies have shown that the wound microbiota in DFU patients typically exhibits higher abundance of Gram-negative bacteria such as *Klebsiella* and *Pseudomonas*, which are closely associated with wound infection and poor healing (Díaz-Velis et al., 2023). Additionally, the microbiota of DFU patients often features reduced diversity, and this loss of diversity is negatively correlated with the severity of foot ulcers (Li YY. et al., 2024). In one study, the gut microbiota of diabetic patients demonstrated significant changes in specific genera compared to healthy controls, particularly a reduction in beneficial bacteria and an increase in pathogenic bacteria, which may be an important factor contributing to DFU development (Jnana et al., 2020). Collect wound samples from DFU patients, divided into three stages: inflammatory phase, proliferative phase, and remodeling phase. Analysis shows that Peptoniphilus, *Lactobacillus*, Prevotella, Veillonella, Dialister, *Streptococcus*, and Ruminococcus were the signature wound microbiota for the inflammatory stage; Anaerococcus, Ralstonia, *Actinomyces*, and Akkermansia were important species for the proliferation stage; and the crucial genera for the remodeling stage were *Enterobacter*, *Pseudomonas*, Sondgrassella, Bifidobacterium, and Faecalibacterium (Li Y. et al., 2024). In another animal experiment, Fufang-zhenzhu-tiaozhi formula treatment increased the content of short chain fatty acids (propionic acid and butyric acid), and inhibited the intestinal flora disorder caused by diabetes, including the growth of Weissella, *Enterococcus* and Akkermansia (Lan et al., 2023). These microbial characteristics not only influence the progression of diabetes but also affect the wound healing process. Therefore, microbiota-targeted intervention strategies may hold significant clinical implications in DFU management.

Studies have shown that gut microbiota dysregulation can influence the progression of diabetes through multiple mechanisms (Deng et al., 2022; Zaky et al., 2021; Liu et al., 2020). First, changes in the gut microbiota lead to a decline in intestinal barrier function and increased intestinal permeability, thereby triggering systemic inflammatory responses (Sechovcová et al., 2024). For example, the proliferation of certain harmful bacteria causes the release of endotoxins, which in turn initiates systemic inflammation—a process recognized as a key inducer of diabetes and its complications (Li et al., 2023). Second, microbial metabolites such as SCFAs play critical roles in regulating immune responses and inflammation. Reduced levels of SCFAs may exacerbate metabolic disorders and inflammation in diabetic patients (Huang et al., 2025). Additionally, gut microbiota dysregulation is associated with endocrine disorders, affecting insulin secretion and action, thus worsening diabetic conditions (Wang J. et al., 2024; Du et al., 2023).

SCFAs are a crucial component of gut microbiota metabolites. Studies have demonstrated that SCFAs play a key role in regulating immune and inflammatory responses, particularly in the progression of DFU. SCFAs inhibit inflammatory reactions by activating G protein-coupled receptors (such as GPR41 and GPR43), reducing the release of pro-inflammatory cytokines and alleviating diabetes-related chronic inflammation (Sechovcová et al., 2024). In diabetic patients, SCFA levels often decrease, which is closely associated with gut microbiota dysregulation and thereby potentially contributes to the occurrence and development of DFU (Li YY. et al., 2024). Additionally, SCFAs enhance intestinal barrier function, preventing endotoxins (such as lipopolysaccharides) from entering the bloodstream and reducing systemic inflammation—a process of significant importance for DFU prevention and treatment (Huang et al., 2025).

In addition to SCFAs, other metabolites such as bile acids and amino acids also play significant roles in the progression of DFU. Bile acids not only play a critical role in fat digestion but also participate in the regulation of inflammatory responses by modulating gut microbiota and influencing metabolic pathways (Dos Santos and Galiè, 2024; Asadi et al., 2022). Studies have found that abnormal bile acid metabolism in diabetic patients is closely associated with the development of DFU, and changes in bile acids may affect the composition of gut microbiota, thereby exacerbating inflammatory reactions (Chen et al., 2024). Furthermore, alterations in amino acid metabolism are also linked to DFU progression. Levels of certain amino acids (such as glutamate and arginine) are significantly elevated in diabetic patients, which is closely correlated with the inflammatory state and tissue damage in DFU (Sutanto et al., 2022). Therefore, regulating the levels of these metabolites may emerge as a new strategy for DFU intervention, alleviating the occurrence and progression of foot ulcers by improving metabolic status (Wang and Wang, 2024).

## 5 Application of STCMP in the treatment of DFU

TCM is a pivotal component of China’s traditional medical heritage, with its theoretical framework grounded in the “Yin-Yang and Five Elements” doctrine, emphasizing the harmonious relationship between the human body and the natural environment. TCM posits that health is a state of balanced Yin and Yang and unobstructed flow of Qi (vital energy) and blood, while diseases arise from the imbalance of Yin and Yang and blockage of Qi and blood circulation. Through syndrome differentiation and personalized treatment, TCM employs STCMP, acupuncture, and other modalities to regulate bodily functions and restore health. In recent years, with the deepening of TCM research, growing evidence indicates that The ability of STCMP (e.g., Jinhuang Powder) and acupuncture can effectively control blood glucose levels and alleviate the incidence of diabetic complications through multi-mechanistic actions, such as modulating gut microbiota, improving metabolism, and enhancing immune function (Chen and Wang, 2021; Feng et al., 2024; Zhang B. et al., 2019).

### 5.1 Application of STCMP in the treatment of DFU

In recent years, A review of studies on STCMP treatment for diabetic foot ulcers over the past decade is summarized in Table 1. Research indicates that commonly used botanical drugs formulas such as Jinhuang Powder have been confirmed to reduce infection rates in diabetic foot ulcer patients. Jinhuang Powder is a powdered preparation composed of botanical drugs such as *Rheum palmatum* L. (Rhubarb, Polygonaceae; official drug name: Dahuang), *Phellodendron chinense* Schneid. (Yellow cypress, Rutaceae; official drug name: Huangbai), *Citrus reticulata* Blanco (Tangerine peel, Rutaceae; official drug name: Chenpi), *Glycyrrhiza uralensis* Fisch. ex DC. (Licorice, Fabaceae; official drug name: Gancao). Jinhuang Powder, when applied topically as an aqueous paste (5 g/cm^2^, changed daily) in a multicenter RCT, regulated gut microbiota to increase SCFA production (particularly butyrate) and reduce pro - inflammatory cytokines (IL - 1β, TNF - α) (Ye et al., 2022). Zizhu Ointment is a semi-solid preparation, mainly composed of *Lonicera japonica* Thunb. (Honeysuckle, Caprifoliaceae; official drug name: Jinyinhua), *G. uralensis* Fisch. ex DC. (Licorice, Fabaceae; official drug name: Gancao), *Paeonia lactiflora* Pall. (Peony root, Paeoniaceae; official drug name: Baishao), and petroleum jelly (base). Reserach has shown that Zizhu ointment may promote the healing of DFU by activating the Wnt/β-catenin signaling pathway with low expression. Simiao Yong’an Decoction (SYD) is a classic TCM formula for treating DFU, consisting of four plant medicines: *L. japonica* Thunb. (Honeysuckle, Caprifoliaceae; official drug name: Jinyinhua), *Scrophularia ningpoensis* Hemsl. (Radix scrophulariae, Scrophulariaceae; official drug name: Xuanshen), *Angelica sinensis* (Oliv.) Diels (Danggui, Apiaceae; official drug name: Danggui), and *G. uralensis* Fisch. ex DC. (Licorice, Fabaceae; official drug name: Gancao) (Peilin and Bao, 2025). Yanan Zhao et al. (2017) showed that it can upregulate the protein and mRNA expression levels of β - catenin and regenerative stem cell protein 3 (Rspo-3) in diabetes wound tissue, and downregulate the expression of GSK-3 β. These findings suggest that SYD promotes wound healing potentially via activation of the Wnt/β-catenin signaling pathway. Notably, Naoxintong capsule (NXT), a traditional Chinese medicine commonly used in cardiovascular and cerebrovascular disorders, has emerged as a potential therapeutic agent for DFU. Preclinical studies demonstrate that NXT significantly accelerates wound healing in type 2 diabetic mice by promoting granulation tissue formation, re-epithelialization, and angiogenesis, with underlying mechanisms involving activation of the PI3K/AKT/eNOS signaling pathway (Song and Chen, 2019). Furthermore, when combined with modern medical research methodologies, the efficacy of STCMP in treating diabetic foot ulcers has been evaluated through randomized controlled trials (RCTs). Shengji ointment is a semi-solid preparation made from *Angelica dahurica* (Fisch. ex Hoffm.) Benth. et Hook. f. (Taiwan angelica root, Apiaceae; official drug name: Baizhi), *Dracaena cochinchinensis* (Lour.) S.C.Chen (Dragon’s blood, Asparagaceae; official drug name: Xuejie), *Cinnamomum camphora* (L.) J.Presl (Borneol, Lauraceae; official drug name: Bingpian), and *Angelica sinensis* (Oliv.) Diels (Chinese angelica, Apiaceae; official drug name: Danggui). Results have shown that Shengji ointment demonstrates significant advantages in improving patients’ quality of life and reducing the recurrence rate of foot ulcers (Zhao Y. et al., 2023; Yang et al., 2022).

**TABLE 1:** Research on the treatment of diabetic foot with STCMP.

References	STCMP	Type of STCMP	Plant source/Composition	Type of study	Research model	Signaling pathway	Mechanisms	Results
Zhang et al. (2024a)	Huangqi Guizhi Wuwu Decoction	Botanical drugs formula	*Astragalus membranaceus* Bunge (Huangqi), *Cinnamomum cassia* (L.) J. Presl (Guizhi), *Paeonia lactiflora* Pall. (Baishao), *Zingiber officinale* Roscoe (Shengjiang), *Glycyrrhiza uralensis* Fisch. ex DC. (Gancao)	*In vivo*	Mice with DPN 3.5 g/kg treatment for 8 weeks	——	GSH↑, SOD↑, IL-10↑, MDA↓, IL-1β↓	The regulation of gut microbiota and lipid metabolism by HGWD may be the mechanism for improving DNP in mice
Fan et al. (2021b)	Procyanidin B2	Isolated metabolite	Extracted from *Vitis vinifera* L. (Putao)	*In vivo In vitro*	STZ-induced diabetic mice EpSCs in healthy newborns 10 mg/kg treatment for 10 days	Nrf2	Nrf2↑, CAT↑, NQO1↑,VEGF↑, ROS↓, 4-HNE↓, MDA↓	Procyanidin B2 treatment can accelerate wound healing and increase angiogenesis in diabetes mice
Sun et al. (2020)	PF	Isolated metabolite	Bioactive constituent of *Paeonia lactiflora* Pall. (Baishao)	*In vivo In vitro*	STZ-induced diabetic rats HaCaT cells 15 mg/kg or 30 mg/kg once a day for 16 consecutive days	HO-1/Nrf2	Nrf2↑, HO-1↑, collagen↑, CD31^+^↑, Ki67^+^↑, SOD↑, GSH↑, VEGF↑, TGF-β1↑ MDA↓, ROS↓	PF treatment improves wound healing in diabetes
Zhang et al. (2020)	Danhuang powder	Botanical drugs formula	*Salvia miltiorrhiza* Bunge (Danshen), *Scutellaria baicalensis* Georgi (Huangqin), *Paeonia lactiflora* Pall. (Baishao), *Rheum palmatum* L. (Dahuang)	*In vivo*	STZ-induced diabetic rats 10 mg/kg treatment for 28 days	TGF-β/Smad3	TGF-β↑, Smad3↑, PCNA↑	Danhuang Powder may promote wound healing of diabetes foot ulcers by activating TGF-β1/Smad3 signaling pathway
Song and Chen (2019)	NXT	Botanical drugs formula	*Astragalus membranaceus* Bunge (Huangqi), *Salvia miltiorrhiza* Bunge (Danshen), *Angelica sinensis* (Oliv.) Diels (Danggui), *Bupleurum chinense* Franch. (Chaihu), *Glycyrrhiza uralensis* Fisch. ex DC. (Gancao)	*In vivo*	Type 2 diabetes mice 70 mg/kg treatment for 17 days	PI3K/AKT; VEGF/eNO3	VEGF↑, p- PI3K↑, p- AKT↑, p- eNO3↑	NXT can significantly accelerate wound healing and increase the granulation tissue formation, re-epithelialization and angiogenesis
Fei et al. (2019)	Shixiang Plaster	Botanical drugs formula	*Boswellia carteri* Birdw. (Chenxiang), *Aquilaria sinensis* (Lour.) Spreng. (Ruixiang), *Cinnamomum cassia* (L.) J. Presl (Guizhi), *Zingiber officinale* Roscoe (Shengjiang), *Glycyrrhiza uralensis* Fisch. ex DC. (Gancao)	*In vivo*	STZ-induced diabetic rats Apply 2 mm thickness locally on the wound and treat for 14 days	RAGE/NF-kappaB; VEGF/VCAM-1/eNOS	VEGF↑, CD34↑, eNOS↑, NF-κB↓ p65↓, AGEs↓, RAGE↓, VCAM-1↓	Shixiang plaster promoted healing in a rat model of diabetic ulcer through the RAGE/NF-kappaB and VEGF/VCAM-1/eNOS signaling pathways
Cao et al. (2019)	Cycloastagalol (CAG)	Isolated metabolite	Hydrolytic product of astragalosides from *Astragalus membranaceus* Bunge (Huangqi)	*In Vitro*	EpSCs Cultivate with different concentrations of CAG (0.3, 1, and 10 μ M) for 7 days	Wnt/β-catenin	TERT↑, β-catenin↑, c-Myc↑	CAG not only promoted the proliferation and migration ability of EpSCs but also increased the expression levels of TERT, β-catenin, c-Myc
Li et al. (2019)	Zizhu Ointment	Botanical drugs formula	*Lonicera japonica* Thunb. (Jinyinhua), *Glycyrrhiza uralensis* Fisch. ex DC. (Gancao), *Paeonia lactiflora* Pall. (Baishao), *Scutellaria baicalensis* Georgi (Huangqin), and petroleum jelly (base)	*In vivo*	Patients with DFU Apply 2 mm thickness locally on the wound and treat for 14 days	Wnt/β-catenin	β-catenin↑, C-myc↑, Rspo-3↑ GSK-3β↓	Zizhu ointment may promote the healing of diabetes foot ulcers by activating the Wnt/β-catenin signaling pathway with low expression
Huang et al. (2019)	epigallocatechin gallate	Isolated metabolite	Polyphenol from *Camellia sinensis* (L.) O. Ktze. (Cha ye)	*In vivo*	STZ-induced diabetic mice	Notch; Nrf2/HO-1	Nrf2↑, HO-1↑, NQO1↑ ICAM-1↓, VCAM-1↓, Caspase 12↓	EGCG improves wound healing in diabetes by targeting Notch signaling pathway
Li et al. (2018)	Hesperidin	Isolated metabolite	*Citrus reticulata* Blanco (Chenpi)	*In vivo*	STZ-induced diabetic rats Hesperidin (25, 50 and 100 mg/kg, p.o.) was administered for 21 days	TGF-ß/Smads; Ang-1/Tie-2	TGF-β↑, Smad2↑, Smad3↑, SOD↑, GSH↑, HYP↑, VEGF-c↑, Ang-1↑, Tie-2↑, MDA↓, NO ↓	Hesperidin accelerates angiogenesis and angiogenesis to enhance wound healing of chronic diabetes foot ulcers
Chen et al. (2018)	Baicalin	Isolated metabolite	Bioactive flavonoid from *Scutellaria baicalensis* Georgi (Huangqin)	*In vivo In vitro*	STZ-induced diabetic mice 50 mg/kg treatment for 28 days HUVECs Cultivate with Baicalin at a concentration of 50 μM for 3 days	Akt/GSK3B/Fyn; Nrf2/HO-1	n-Nrf2↑, NQO1↑, NQO2↑, HO-1↑, CAT↑, SOD2↑, p-Akt↑, p-GSK 3β↑, c-Nrf2↓, Bax/Bcl-2↓, c-Caspase 3↓, 3-NT+↓, IL-1β↓, IL-6↓, IL-8↓, TNF-α↓, n-Fyn↓	The endothelial protective effect of baicalin under hyperglycemic conditions may be partially attributed to its activation of Nrf2, downregulation of ROS, and inflammation
Zhao et al. (2017)	Simiao Yong’an Decoction	Botanical drugs formula	*Lonicera japonica* Thunb. (Jinyinhua), Scrophularia ningpoensis (Xuanshen), *Angelica sinensis* (Oliv.) Diels (Danggui), *Glycyrrhiza uralensis* Fisch. ex DC. (Gancao)	*In vivo*	STZ-induced diabetic rats 30 mg/kg treatment for 28 days	Wnt/β-catenin	β-catenin↑,GSK-3β↓	Simiao Yong’an Decoction could promote the healing of DU possibly by regulating Wnt/β-catenin signaling pathway
Liu et al. (2016)	Qizhi Jiangtang Capsule	Botanical drugs formula	*Astragalus membranaceus* Bunge (Huangqi), *Cinnamomum cassia* (L.) J. Presl (Guizhi), *Glycyrrhiza uralensis* Fisch. ex DC. (Gancao)	*In vivo*	STZ-induced diabetic rats 2.24 g/kg treatment for 56 days	——	VEGF↑, p-ERK↓	Qizhi Jiangtang capsule may promote the healing of skin ulcer in diabetes rats by regulating the expression of VEGA and p-ERK protein

CAG, cycloastagalol; DFU, diabetes foot ulcer; EpSCs, Epidermal stem cells; HUVECs, Human umbilical vein endothelial cells; PF, paeoniflorin; STZ, streptozotocin; eNos, Endothelial nitric oxide synthase; HO-1, Heme oxygenase-1; Rspo-3, R-spondin 3; VEGF, vascular endothelial growth factor; NQO1, NAD(P)H Quinone Dehydrogenase 1.

Paeoniflorin (PF), a water - soluble monoterpenoid glycoside metabolite of *P. lactiflora* Pall. (Peony root, Paeoniaceae; official drug name: Baishao), including anti-inflammatory, antioxidant, analgesic, hypoglycemic, and neuroprotective activities (Zhang and Wei, 2020). Sun et al. demonstrated that PF significantly attenuated wound inflammation in DFU rats, with marked downregulation of proinflammatory cytokines IL-1β, IL-18, and TNF-α in PF-treated DFU rat models (Sun et al., 2021). Cycloastagalol (CAG), a triterpenoid saponin hydrolytic product derived from *A. membranaceus* Bunge (Milkvetch root, Fabaceae; official drug name: Huangqi), exhibits a broad spectrum of pharmacological activities, including anti-aging, anti-apoptotic, and anti-inflammatory effects (Li M. et al., 2020). Emerging evidence has demonstrated that CAG not only significantly enhances the proliferative and migratory capacities of human epidermal stem cells (EpSCs) but also upregulates the expression levels of telomerase reverse transcriptase (TERT), β-catenin, and C-Myc. Notably, the CAG-mediated promotion of EpSC proliferation and migration was completely abrogated in TERT- and β-catenin-silenced cell models (Cao et al., 2019). Additionally, *Angelica sinensis* (Oliv.) Diels (Danggui, Apiaceae; official drug name: Danggui) and *Salvia miltiorrhiza* Bunge (Danshen root, Lamiaceae; official drug name: Danshen) can regulate the intestinal microbiota, increase SCFAs, reduce proinflammatory cytokines (IL-1 β, TNF - α), and enhance the integrity of the intestinal barrier through tight junction proteins (ZO-1, claudin-1), thus preventing harmful substances from entering the blood, reducing systemic inflammatory response, and alleviating the symptoms of diabetes foot ulcers (Lu et al., 2024; Hu and Wu, 2023; Zhang L. et al., 2024; Huang H. et al., 2024). STCMP can also modulate the metabolites of gut microbiota, promoting host immune regulation and metabolic balance, thereby improving diabetes-related metabolic disorders and inflammatory states (Chen and Wang, 2021). Liang et al. reported that external treatments with STCMP, such as botanical drugsl foot baths and topical applications, can effectively relieve pain and discomfort in diabetic foot ulcers, promote local blood circulation, and improve patients’ quality of life (Liang et al., 2024). However, current studies still suffer from issues such as small sample sizes and less rigorous study designs. More high-quality clinical trials are needed in the future to validate the efficacy and safety of STCMP in the treatment of diabetic foot ulcers (Fan S. et al., 2021).

### 5.2 Application of acupuncture in the treatment of DFU

Acupuncture, as an adjunctive therapy, has been gaining increasing attention in the application of diabetes and its complications, particularly in the management of diabetic foot ulcers (Jung et al., 2023). Acupuncture encompasses diverse therapeutic modalities, including encircling needling, Bangci (focal center-side needling), auricular acupuncture, pestle needling therapy, electroacupuncture, moxibustion and traditional acupuncture (Zhang et al., 2015). Multiple clinical studies have demonstrated that acupuncture can effectively improve symptoms in diabetic patients and reduce the incidence of complications (as shown in Table 2). One study investigated 18 patients with diabetic foot syndrome who underwent acupuncture treatment, and results showed significant improvement in microcirculatory parameters following intervention, suggesting that acupuncture may promote healing by enhancing local blood flow (Valentini et al., 2024). Following auricular acupuncture treatment in type 2 diabetes mellitus (DM) patients, significant improvements in lower extremity blood flow and elevation of plantar skin temperature were observed, collectively indicating a potential preventive effect against diabetic foot (DFU) (Bacelar de Assis et al., 2021). Wei et al. found that both encircling needling and Bangci (focal center-side needling) can promote wound healing in DM mice by increasing local blood perfusion, and the therapeutic effect of circumferential acupuncture is better than Bangci (Wei et al., 2020). In an animal study, Kan et al. found that moxibustion can promote wound healing by promoting collagen fiber growth and cell proliferation (Kan et al., 2019).

**TABLE 2:** Research on the treatment of diabetic foot with acupuncture.

References	Intervention methods	Type of study	Participants	Research model	Mechanisms	Results
Huang et al. (2024b)	Acupuncture	RCT	E:30 C:30	Patients with Wagner grade 0 diabetic foot 5 min x2/week for 8 weeks	——	Acupuncture artery technique at Zusanli (ST 36) could effectively improve the clinical symptoms of patients with Wagner grade 0 diabetic foot
Yuan et al. (2024)	EA	*In vivo*	E:8 C:8	STZ-induced diabetic rats 20 min x6/week for 6 weeks	Sirt1↑, PGC-1α↑, TFAM ↑	EA treatment can improve and repair the function of damaged peripheral nerves in DPN rats
Wang et al. (2024a)	EA	*In vivo*	E:10 C:10	STZ-induced diabetic rats 15 min x3/week for 8 weeks	LC3-II↑, PTEN↑, PI3K↓	EA inhibits the activation of PI3K, increases autophagy levels, and protects podocytes
Dietzel et al. (2023)	TA	RCT	E:31 C:31	Patients with type II diabetes and symptoms of neuropathy in the lower limbs 25 min x3/week for 8 weeks	——	Acupuncture and moxibustion can significantly and persistently reduce DPN related complaints with good tolerance and slight side effects
Bacelar de Assis et al. (2021)	AA	RCT	E:22 C:22	Patients with type 2 diabetes 20 min x5/week	——	AA effectively improves circulation conditions and plantar temperature
Wang et al. (2021)	EA	*In vivo*	E:10 C:10	STZ-induced diabetic rats 20 min x6/week for 5 weeks	GLO1↑, AGEs↑, RAGE↑, IL-1β↓, IL-6↓, TNF-α↓	EA may exert therapeutic effects on DNP by regulating metabolism
Wei et al. (2020)	EN, Bangci	*In vivo*	E:8 C:8	STZ-induced diabetic mice 30 min x7/week for 2 weeks	——	Both EN and Bangci can promote the skin wound healing by increasing the blood perfusion in diabetic mice
Kan et al. (2019)	Moxibustion	*In vivo*	E:14 C:14	SD male rats 25 min x1/day for 6 days	IL-10↑, TGF-β↑ VEGF↑	Moxibustion can promote wound healing by promoting collagen fiber growth and cell proliferation
Wang et al. (2018)	PN	RCT	E:66 C:66	Patients of high-risk diabetic foot 30 min x7/week for 4 weeks	——	The PN therapy improves the sensory nerve function of the foot in the patients of high-risk diabetic foot

C, Control group; E, experimental group; EA: electroacupuncture; TA: traditional acupuncture; AA: auricular acupuncture; EN: encircling needling; PN: pestle needling; DPN: diabetic peripheral neuropathy; AGEs: Advanced glycation end products; GLO1: Glyoxalase-1; RAGE: Receptor for AGEs.

Additionally, acupuncture exhibits unique advantages compared with traditional treatment methods (such as pharmacological therapy and surgical intervention) in the management of diabetic foot ulcers. Acupuncture can effectively reduce patients’ pain scores and demonstrates better efficacy in promoting wound healing. For instance, studies have shown that acupuncture combined with STCMP treatment achieves a higher response rate in improving diabetic foot ulcers compared with conventional pharmacological therapy alone (Tanasov et al., 2025). Additionally, acupuncture has few side effects and is well-tolerated by patients, which makes it a safe and effective alternative treatment option (Huang Z. et al., 2024; Dietzel et al., 2023). Mechanistically, acupuncture may exert its therapeutic effects by downregulating the protein expression of proinflammatory cytokines tumor necrosis factor-α (TNF-α) and interleukin-1β (IL-1β), while concurrently promoting neovascularization and enhancing fibroblast recruitment/activity in the wound microenvironment (Park et al., 2012). These research findings provide strong evidence for the application of acupuncture in diabetic foot ulcers, indicating that as a safe and effective treatment option, it is warranted for further clinical promotion.

## 6 Mechanism of STCMP regulating intestinal microflora in treating DFU

### 6.1 Regulating inflammatory response to improve foot symptoms

Inflammation is a critical factor in the occurrence and progression of DFU. Studies show that many STCMP can suppress the release of inflammatory cytokines through multiple signaling pathways, thereby alleviating inflammation. For example, botanical drugs such as *A. membranaceus* Bunge (Milkvetch root, Fabaceae; official drug name: Huangqi) and Dashen have been confirmed to reduce levels of inflammatory cytokines (e.g., IL-6 and TNF-α) in intestinal and skin tissues, thereby mitigating diabetes-induced inflammation (Zhang Q. et al., 2024). Significantly, astragaloside IV upregulates VEGF expression via PI3K/AKT activation, while gallic acid suppresses TNF-α and IL-1β secretion by blocking IκB phosphorylation in the NF-κB pathway (Zhang X. et al., 2024). Additionally, TCM regulates macrophage polarization by promoting the generation of M2-type macrophages, which helps inhibit excessive inflammation and accelerate wound healing (Zhao X. et al., 2023). This multi-target anti-inflammatory effect gives TCM potential application value in DFU treatment.

Research indicates that gut microbiota dysregulation triggers chronic inflammation, leading to diabetes and its complications. STCMP (e.g., Jinhuang Powder, Shengjiang Xiexin Decoction) ameliorate gut microbiota dysbiosis by enriching beneficial bacteria (*Lactobacillus*, Bifidobacterium) and inhibiting pathogens. A study by Ma et al. found that certain STCMP inhibit the release of pro-inflammatory cytokines and enhance the expression of anti-inflammatory factors, thereby restoring intestinal immune balance. The specific mechanisms may involve regulating the integrity and function of intestinal epithelial cells, promoting intestinal barrier repair, reducing intestinal permeability, and decreasing the release of endogenous pro-inflammatory substances (Ma et al., 2023). Furthermore, Huangqi - Guizhi - Wuwu - Decoction can further modulate host immune responses and enhance the body’s anti-inflammatory capacity by influencing gut microbial metabolites such as SCFAs (Zhang K. et al., 2024).

Acupuncture is also recognized as an effective anti-inflammatory treatment. Studies have shown that acupuncture can promote the production of SCFAs, which significantly reduce inflammatory levels in diabetic patients by regulating immune responses and decreasing the release of inflammatory mediators (Tanasov et al., 2025; Yang et al., 2024). Specifically, acupuncture inhibits the activation of inflammatory cells and the release of cytokines by activating the body’s anti-inflammatory pathways, such as the vagus nerve-antihflammatory pathway, thereby alleviating local and systemic inflammatory responses (Shen et al., 2024). In a study on diabetes rats, acupuncture at specific acupoints, such as Zusanli (ST36), “Sanyinjiao” (SP6), “Pishu” (BL20), and “Shenshu” (BL23) can stimulate sensory nerves, and then regulate intestinal microbiota (Ai et al., 2025).

Additionally, acupuncture can further reduce inflammation by improving blood circulation and promoting the repair of damaged tissues. Current research confirms that acupuncture modulates the gut microbiota to significantly influence inflammatory responses, thereby improving symptoms of diabetic foot ulcers (Bragg et al., 2024). Diabetic patients typically exhibit high inflammatory levels, which are closely linked to gut microbiota dysregulation. Acupuncture stimulates specific acupoints to promote immune system balance and reduce the release of inflammatory factors such as TNF-α and IL-6 (Ynag et al., 2021; Xie et al., 2021). The reduction of these factors helps alleviate local and systemic inflammatory responses, thereby promoting foot ulcer healing. These mechanisms highlight the important clinical significance of acupuncture in DFU treatment.

### 6.2 Promoting healing by improving microcirculation

We now specify that *A. membranaceus* Bunge (Milkvetch root, Fabaceae; official drug name: Huangqi) plays a role through its metabolite cycloastagalol (CAG). CAG enhances the proliferation of epidermal stem cells through Wnt/β - catenin pathway, thus promoting wound repair in diabetes (Cao et al., 2019). Dang-Gui-Si-Ni decoction is a classic traditional Chinese medicine formula composed of six plant medicines: *Angelica sinensis* (Oliv.) Diels (Danggui, Apiaceae; official drug name: Danggui), *Cinnamomum cassia* (L.) J. Presl (Cassia twig, Lauraceae; official drug name: Guizhi), *P. lactiflora* Pall. (Peony root, Paeoniaceae; official drug name: Baishao), *Asarum heterotropoides* F. Schmidt (Manchurian wild ginger, Aristolochiaceae; official drug name: Xixin), *Tetrapanax papyrifer* (Hook.) K. Koch (Rice paperplant pith, Araliaceae; official drug name: Tongcao), *G. uralensis* Fisch. ex DC. (Licorice, Fabaceae; official drug name: Gancao) (Xinyue Niu et al., 2024). Research has shown that Dang-Gui-Si-Ni decoction can significantly increase the abundance of beneficial intestinal bacteria and improve microbial diversity by regulating the expression of AGEs/RAGE/TGF - β/Smad2/3, thereby enhancing overall metabolic function and promoting wound healing in DFU (Zhang et al., 2024e). The botanical drug formulas allows them to act on multiple targets simultaneously, enhancing therapeutic effects. For example, Shengjiang Xiexin Decoction is a decoction composed of *Zingiber officinale* Roscoe (Ginger, Zingiberaceae; official drug name: Shengjiang), *Panax ginseng* C.A. Mey. (Ginseng, Araliaceae; official drug name: Renshen), *Pinellia ternata* (Thunb.) Breit. (Pinellia tuber, Araceae; official drug name: Banxia), *Scutellaria baicalensis* Georgi (Baikal skullcap root, Lamiaceae; official drug name: Huangqin) and *G. uralensis* Fisch. ex DC. (Licorice, Fabaceae; official drug name: Gancao). It can modulate gut microbiota to promote SCFAs production, thereby improving diabetes-related inflammation and insulin resistance (Yu et al., 2024). Research indicates that gut microbiota ferment dietary fiber to produce SCFAs, which not only serve as energy sources but also regulate inflammatory responses and metabolic processes by activating G protein-coupled receptors (Li YJ. et al., 2020). TCM components such as flavonoids and polyphenols have been found to promote the growth of beneficial bacteria, thereby increasing SCFA production and improving intestinal health and metabolic status (Hu and Wu, 2023).

Additionally, Treatment methods that affect bile acid metabolism to regulate the composition of intestinal microbiota further affect the metabolic function of the host and reduce complications related to diabetes (Lu et al., 2024; Wu et al., 2022; Cai et al., 2022). Notably, The plant metabolites like glycyrrhizic acid and ginsenosides promote skin cell proliferation and migration, thereby accelerating wound healing (Feng et al., 2024). Studies show that TCM also accelerates skin healing by enhancing angiogenesis, improving local blood flow, and promoting collagen synthesis (Zhao X. et al., 2023). These mechanisms provide a theoretical and practical basis for TCM in treating skin complications like DFU.

Improving microcirculation represents a key mechanism by which acupuncture facilitates the healing of DFU. Studies have demonstrated that acupuncture significantly enhances blood flow in the lower limbs and improves microvascular function. This process activates local nerves and blood vessels to promote blood circulation, thereby enhancing nutritional supply to tissues and the clearance of metabolic waste (Huang Z. et al., 2024). Improved microcirculation not only accelerates wound healing but also reduces the risk of infection and the incidence of DFU complications (Lee et al., 2020; Valentini et al., 2024). Related research shows that acupuncture leads to significant improvements in foot temperature and hemodynamic parameters in patients, providing a favorable physiological foundation for ulcer healing (Valentini et al., 2024; Gu et al., 2022).

Besides, acupuncture regulates the host’s metabolic state by influencing gut microbiota composition. Specifically, it promotes the production of SCFAs, which serve not only as an energy source for intestinal cells but also affect systemic metabolism by suppressing appetite and improving insulin sensitivity (Zhang et al., 2024f). Acupuncture may further modulate the release of inflammatory cytokines by regulating gut microbial metabolites, thereby alleviating insulin resistance and lipid metabolism disorders (Luo et al., 2025). Certain studies indicate that acupuncture reduces intestinal inflammation, thereby improving metabolic syndrome-related symptoms such as obesity and hyperglycemia (Yin et al., 2025). Notably, acupuncture also stimulates the proliferation and migration of fibroblasts and keratinocytes, which are critical for wound healing (Wang KX. et al., 2024; Zhao CJ. et al., 2023). As a non-pharmacological therapy, acupuncture demonstrates potential to improve metabolic health through gut microbiota regulation while fundamentally enhancing skin healing capacity. This multi-mechanistic approach offers innovative perspectives for the treatment of diabetes and its complications (Zhang et al., 2023).

### 6.3 Promote the improvement of intestinal barrier function

The integrity of the intestinal barrier is crucial for maintaining gut health, while gut microbiota dysregulation can impair barrier function, triggering a series of metabolic diseases (Tian et al., 2022). TCM strengthens the tight junctions of intestinal epithelial cells and promotes barrier repair by modulating gut microbiota. For example, *A. membranaceus* Bunge (Milkvetch root, Fabaceae; official drug name: Huangqi) upregulate the expression of tight junction proteins such as ZO-1 and claudin-1, thereby enhancing intestinal barrier integrity (Luo et al., 2024). Additionally, *G. uralensis* Fisch. ex DC. (Licorice, Fabaceae; official drug name: Gancao) improves gut microbial metabolites to increase the thickness of the intestinal mucus layer, further protecting intestinal epithelial cells and preventing the invasion of harmful substances (Luo et al., 2024).

Baizhu shaoyao decoction is a soup made from *Atractylodes macrocephala* Koidz. (Largehead atractylodes rhizome, Asteraceae; official drug name: Baizhu), *P. lactiflora* Pall. (Peony root, Paeoniaceae; official drug name: Shaoyao), and Glycyrrhiza uralensis Fisch. ex DC. (Licorice, Fabaceae; official drug name: Gancao). A study by Wei et al. demonstrated that Baizhu shaoyao decoction alleviates depressive and intestinal symptoms by regulating brain-gut peptide expression and restoring intestinal barrier function via the forkhead box O signaling pathway (Wei et al., 2024). Notably, *A. membranaceus* Bunge (Milkvetch root, Fabaceae; official drug name: Huangqi) and *Panax ginseng* C.A. Mey. (Ginseng root, Araliaceae; official drug name: Renshen) have been confirmed to enhance gut microbial diversity and inhibit the growth of harmful bacteria (Shan et al., 2024). These mechanisms not only help improve symptoms of DFU but also provide new insights for the prevention and treatment of related diseases.

Acupuncture’s role in regulating intestinal function, particularly among DFU patients, is garnering increasing attention. Studies indicate that acupuncture improves gut motor function and microbial community balance by stimulating specific acupoints, promoting intestinal health and thereby enhancing the skin’s self-repair capacity (Xu et al., 2022). This mechanism may be associated with acupuncture’s regulation of the enteric nervous system, as it activates the vagus nerve to enhance intestinal blood flow, improve nutrient absorption, and boost metabolic function (Huang Z. et al., 2024). Additionally, acupuncture reduces intestinal permeability in diabetic patients by modulating intestinal inflammatory responses, thereby alleviating diabetes-related complications such as foot ulcers (Valentini et al., 2024). A study by Hao et al. demonstrated that manual acupuncture benignly regulates gut microbiota dysbiosis, significantly reduces intestinal inflammation, and effectively mitigates intestinal mucosal barrier damage in APP/PS1 mice—with effects comparable to probiotics (Hao et al., 2022). Therefore, acupuncture can serve not only as an adjunctive therapy for DFU but also as a strategy to reduce overall risks in diabetic patients by improving intestinal function.

## 7 Future prospect of treating DFU based on the regulation of intestinal flora by TCM

In a RCT, patients in the acupuncture treatment group can significantly enhance the treatment effect without side effects (Xia et al., 2010). In a randomized controlled trial of DFU, the control group received gentamicin alone, while the treatment group received external application of Jinhuang powder combined with gentamicin. After 6 weeks of treatment, compared with the control group, the average wound healing time of the treatment group was shortened by nearly 7 days, and the effective rate reached 92% (Xiaobin Cui and Zhenli, 2011). In Liu et al.'s study (Liu, 2024), the effective rate and average wound healing time of the combination of Ruyi Jinhuang powder and Western medicine in the treatment of DFU were significantly better than those of the control group, which is consistent with the previous research results of Cui et al. Acupuncture, for instance, requires no complex equipment and can be administered by primary care providers after standardized training, ensuring wide applicability. Similarly, TCM formulas can often be locally prepared using readily available botanical drugs, reducing reliance on complex supply chains.

STCMP and acupuncture belong to one of Chinese medicine treatment methods.Despite the promising potential of STCMP and acupuncture in the treatment of DFU, their clinical application still faces numerous challenges. First, the lack of unified treatment standards and guidelines leaves clinicians without clear directions or evidence-based protocols when applying STCMP and acupuncture (Liu et al., 2023). Second, there is varied patient acceptance of STCMP and acupuncture, as some patients may remain skeptical of traditional therapies, affecting treatment compliance. Additionally, the mastery of professional knowledge and skills regarding STCMP and acupuncture is uneven across clinical settings, with some medical institutions lacking relevant training and resources, which restricts the promotion and application of these treatments (Selçuk et al., 2022). Finally, data on the efficacy and safety of STCMP and acupuncture remain insufficient, causing clinicians to hesitate when selecting treatment protocols due to the lack of robust evidence. Therefore, conducting large-scale, multi-center RCTs is of utmost importance. Such studies would not only validate the efficacy and safety of STCMP but also provide a scientific basis for its integration into modern medical practice.

Existing studies on the use of STCMP and acupuncture in DFU treatment, while providing preliminary evidence, have numerous methodological limitations. First, many studies have small sample sizes, calling into question the reliability of their results. For example, although some literature mentions the effects of acupuncture on diabetic peripheral neuropathy and shows certain efficacy, the insufficient sample size makes it difficult to generalize findings to a broader patient population (Meyer-Hamme et al., 2021); while (Yanan Zhao et al., 2017) demonstrated that SYD activates the Wnt/β-catenin pathway in diabetic ulcer rats, we note that the study lacked a dose-response analysis, which limits conclusions about optimal therapeutic concentrations. Similarly, in the clinical trial by Bacelar de Assis et al. (2021) on auricular acupuncture for diabetic foot, we highlight that the small sample size (n = 44) and short follow-up period (8 weeks) restrict the generalizability of their findings on improved peripheral circulation. Second, many studies lack RCTs designs, failing to effectively exclude potential biases and undermining the credibility of results. Additionally, The lack of standardization in the study of botanical drugs metabolites and dosage control makes it difficult to compare and synthesize the results of different studies. Moreover, the reports on botanical drugs extraction methods are inconsistent (for example, Honeysuckle: water extract and ethanol extract in the study) (Xing et al., 2022). Finally, many studies do not adequately account for patient-specific differences such as age, gender, and diabetes type, all of which may influence the evaluation of treatment outcomes.

To better evaluate the role of TCM and acupuncture in DFU treatment, future research should focus on several key aspects. First, it is recommended to conduct large-scale RCTs to enhance the reliability and generalizability of research findings (Fu et al., 2020). These studies should incorporate multi-center designs to ensure sample diversity and representativeness (Fan et al., 2024). Second, research should emphasize standardization of botanical drugs metabolites and dosage control to facilitate comparison and integration of results across different studies. Additionally, future studies should account for patient-specific differences, exploring how different patient groups respond to TCM and acupuncture to develop personalized treatment protocols (Xing et al., 2022). Although there is evidence to support the upregulation of SCFA in traditional Chinese medicine, quantitative insights are still limited. Finally, by integrating modern biotechnology with TCM theories, investigations into how TCM components influence host metabolism through modulating gut microbiota could provide new strategies for DFU treatment (Zhu et al., 2024). We will continue to conduct in-depth research on the mechanism of gut skin axis crosstalk, and combine multi omics (metagenomics, metabolomics) to elucidate how traditional Chinese medicine regulates microbial metabolites (such as SCFA, bile acids) and downstream pathways (such as Nrf2, PI3K/AKT) in human DFU samples. Meanwhile, researchers must also address the long-term effects of TCM on gut microbiota and its safety profile to ensure the efficacy and safety of clinical applications (Li X. et al., 2024).

## 8 Conclusion

As research on the gut-skin axis deepens, an increasing body of evidence indicates that gut microbiota and immune responses play pivotal roles in the initiation and progression of DFU. Therefore, modulating intestinal health and improving skin condition will emerge as critical strategies in the management of DFU.

In the perspectives and findings of different studies, the effectiveness of STCMP and acupuncture is closely linked to their unique mechanisms of action. STCMP (e.g., Jinhuang Powder, Shengjiang Xiexin Decoction) ameliorate gut microbiota dysbiosis by enriching beneficial bacteria (*Lactobacillus*, Bifidobacterium) and inhibiting pathogens, while acupuncture modulates neuroendocrine and immune responses by stimulating specific acupoints. The combination of these two not only complements each other’s shortcomings but also creates a synergistic effect, thereby enhancing clinical efficacy.

However, in research and clinical applications, we must also exercise caution. Varied study designs, sample selection, and intervention protocols may lead to divergent results. Therefore, future research should place greater emphasis on standardized research methodologies and conduct large-scale RCTs to validate the true efficacy and safety of the combined application of STCMP and acupuncture. In addition, Future studies should focus on identifying specific microbial taxa (e.g., butyrate-producing bacteria like Faecalibacterium prausnitzii) modulated by TCM botanical drugs (e.g., *A. membranaceus* Bunge). Combining metagenomic sequencing with metabolomics could reveal how botanical drugs metabolites (e.g., astragaloside IV) reshape microbial metabolic pathways (e.g., SCFA synthesis) to enhance skin repair.

In summary, STCMP and acupuncture demonstrates broad prospects in the treatment of DFU. Through further research, we expect to develop more effective treatment strategies based on the modulation of the gut-skin axis, thereby improving patients’ quality of life.

**FIGURE 2:**
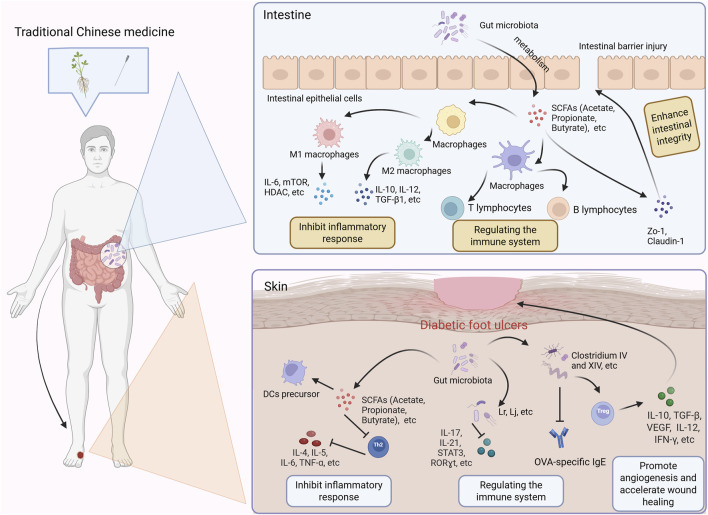
Potential mechanism of traditional Chinese medicine regulating intestinal flora in treating diabetes foot ulcer. SCFAs: Short-chain fatty acids; DCs: Dendritic Cells.
